# Enhanced Degradation of Levofloxacin through Visible-Light-Driven Peroxymonosulfate Activation over CuInS_2_/g-C_3_N_4_ Heterojunctions

**DOI:** 10.3390/nano14010074

**Published:** 2023-12-26

**Authors:** Xin Zhong, Meihuan Ji, Wenxin Wu, Caicai Lu, Wenping Liu, Fubin Jiang

**Affiliations:** 1 Experimental and Practical Innovation Education Center, Beijing Normal University at Zhuhai, Zhuhai 519087, China; meihuanji@bnu.edu.cn (M.J.); wxw1018@outlook.com (W.W.); lucaicai@bnu.edu.cn (C.L.); liuwp@bnu.edu.cn (W.L.); 2 Department of Environmental Engineering and Science, Beijing Normal University at Zhuhai, Zhuhai 519087, China

**Keywords:** peroxymonosulfate, visible-light-assisted PMS activation, antibiotics

## Abstract

In this work, the heterojunctions of CuInS_2_ embedded in the g-C_3_N_4_ materials (xCuInS_2_/g-C_3_N_4_, abbreviated as xCIS/GCN) was successfully prepared for peroxymonosulfate (PMS) activation under visible light. The catalysts are characterized by different techniques, such as XRD, FTIR, SEM, TEM, and UV-vis. The unique heterojunction composites can suppress the recombination of photogenerated pairs. The catalytic results showed that the 3CIS/GCN exhibited excellent catalytic levofloxacin (LVF) degradation efficiency, while more than 98.9% of LVF was removed in 60 min over a wide pH range. SO_4_^•−^, O_2_^•−^, OH^•^, and ^1^O_2_ were verified as the main reactive species for LVF degradation via the quenching experiments and electron paramagnetic resonance technology (EPR). The synergetic effect of xCIS/GCN, PMS, and visible light irradiation was discussed. The possible LVF degradation pathway was proposed through byproducts analysis (LC-MS). Moreover, the 3CIS/GCN/vis-PMS system has very low metal leaching. Owing to xCIS/GCN having good properties for PMS activation, it has potential applications for LVF or other hazardous pollutants degradation.

## 1. Introduction

During the past few decades, the problems of environmental pollution and energy shortages have been the most severe threat to continuous development worldwide [1,2,3,4]. To overcome these problems, especially in wastewater treatment processes, it is particularly crucial to find new technologies that can achieve clean, efficient, and economic development in contrast to conventional treatment methods, which struggle with excessive chemical consumption, low energy efficiency, and complicated operation procedure [5,6,7,8]. Among various industrial wastewaters, the excess discharge of antibiotics in the environment has aroused severe concerns due to the residual antibiotics in the environment, leading to drug-resistant problems in everyday life [9,10,11]. Levofloxacin (LVF) is one of the most widely used antibiotics for disease control in medical applications [12]. However, residual LVF (usually trace concentrations) can be digested by animals and plants, leading to the contamination of soil, the water matrix, and seafood [13,14]. Hence, it is crucial to find environmentally friendly strategies to eradicate these residues from aqueous water matrices.

Many treatment methods have been applied to degrade various antibiotics, such as the activated sludge method, adsorption method, and advanced oxidation technology [15,16,17]. Among various AOPs, sulfate radical-based advanced oxidation processes (SR-AOPs) have received more attention owing to their simple operation equipment, mild reaction conditions, low cost, long lifespan, and high catalytic efficiency [18,19,20]. In contrast to hydroxyl radicals (1.8–2.7 V vs. NHE), sulfate radicals possessed higher oxidation potential (2.5–3.1 V vs. NHE), longer half-life time, and higher selectivity to the refractory chemicals, which exhibit good potential for refractory contaminants in water [21,22,23]. Moreover, many researchers have shown that other reactive oxygen species could also be detected in the sulfate radical-based AOPs at the activation of peroxymonosulfate (PMS, HSO_5_^−^) and peroxydisulfate (PDS, S_2_O_8_^2−^), such as hydroxyl radicals, superoxide radicals, and/or singlet oxygen, which exhibited satisfactory catalytic performance [24,25,26]. Peroxymonosulfate can be activated via heat, UV light, and transition metals in heterogeneous and homogeneous catalysis systems [27,28,29,30]. Compared to other activation methods, heterogeneous activation has unique advantages due to its simple and easy operation during the experiments, low cost, easy separation from the aqueous solution, and the prevention of secondary metal pollution [31,32,33]. However, some bottleneck issues were also found in the heterogeneous PMS activation process, including metal ion leaching and an inefficient conversion efficiency of the active metal valence state.

Many researchers have confirmed that semiconductor catalyst-induced photocatalytic technology is an effective and environmentally friendly wastewater treatment method owing to its low energy consumption, good stability, and harmless discharge [34,35,36]. Various semiconductors have been studied and applied in photocatalytic processes, such as MOF [37,38,39], g-C_3_N_4_ [40,41,42,43], CuS [44], and ZnO [45]. Graphite carbon nitride (g-C_3_N_4_) has been considered a promising material that possesses a strong band structure, suitable band gap, good adaptability as a precursor, simple synthesis procedure, and suitable physical and chemical properties. However, there are some problems in applying pure GCN in semiconductor photocatalytic processes, such as the quick recombination of photogenerated electron-hole pairs and low efficiency in the degradation rate [46,47,48]. In this case, heterojunctions made of two different materials have received great attention to accelerate photogenerated pair transfer and strengthen the catalyst stability during the processes [49]. As a typical compound of Group-I-III-VI chalcopyrite structures, CuInS_2_ (CIS) showed good potential in photo responsive properties to the visible light, low toxicity, and a suitable band gap [50,51,52]. Combining two materials with different band gaps to make heterojunctions has unique advantages in photocatalytic degradation by accelerating electron-hole transfer [53]. The heterojunctions constructed by CIS and GCN are still rare for the application in the SR-based AOPs.

To obtain satisfactory removal efficiency, several studies have paid great attention to the visible-light-assisted PMS activation process, which can speed up the transfer between photogenerated pairs, leading to better catalytic performance [54,55,56]. There is a synergistic effect between the visible-light-driven photocatalysis process and SR-based heterogeneous AOPs, which intensely improved the catalytic activity in wastewater treatment [57]. The photogenerated pairs can be effectively separated by reacting with PMS, avoiding quick recombination during the reaction process [58]. Nevertheless, the metal state cycle can also be accelerated by the photogenerated pairs.

Consequently, the heterojunctions CIS/GCN were fabricated in visible-light-driven SR-based AOPs for LVF degradation, which has the potential to accelerate the degradation process and electron/hole (e^−^/h^+^) transfer to generate reactive oxygen species. The heterojunctions were prepared through a simple hydrothermal method. The catalytic performance of the xCIS/GCN materials for PMS activation was studied under visible light, using levofloxacin (LVF) as a target pollutant under different parameters. The degradation mechanisms are investigated through radical quenching experiments and EPR analysis. The degradation intermediates are also detected using LC–MS/MS to obtain the LVF degradation pathway. The degradation of LVF in the photocatalysis process by different kinds of photocatalysts was studied and is shown in Table S2. Moreover, the synergistic effect between the visible-light-driven photocatalysis processes and PMS activation was carefully proposed. This work is an attempt to synthesize heterojunctions of CuInS_2_ with g-C_3_N_4_ to enhance the activity of pure GCN materials, which constitutes a new strategy for designing high-performance heterojunction photocatalysts.

## 2. Materials and Methods

### 2.1. Chemicals and Materials

Melamine (C_3_H_5_N_6_, AR), urea (CH_4_N_2_O, AR), copper (II) chloride dihydrate (CuCl_2_·2H_2_O, AR), thioacetamide (C_2_H_5_NS, TAA, AR), indium (III) chloride tetrahydrate (InCl_3_·4H_2_O. AR), levofloxacin (C_18_H_20_FN_3_O_4_, AR), methanol (MeOH, AR), rhodamine B (RhB, AR), Orange II (AR), and phenol (AR) were purchased from Shanghai Aladdin Biochemical Technology Co., Ltd. (Shanghai, China). Tert-butanol (TBA, C_4_H_10_O, AR), peroxymonosulfate (PMS, OXONE, KHSO_5_·HSO_4_·K_2_SO_4_, AR), and ethanol (EtOH, AR) were purchased from Shanghai Yuan-Ye Bio-Technology Co., Ltd. (Shanghai, China). All materials were of analytical grade and used without further purification. All aqueous solutions were prepared with ultrapure water. 

### 2.2. The Synthesis of GCN

At first, 10.0 g of urea and 5.0 g of melamine were mixed and vigorously stirred for 1 h. Then, the mixture was transferred to an autoclave with a Teflon liner and heated at 120 °C for 6 h. The obtained mixture was dried at 70 °C and annealed to 550 °C for 6 h at a heating rate of 5 °C min^−1^ in a muffle furnace. After calcination, the samples were labelled as GCN.

### 2.3. The Synthesis of CIS

The CuInS_2_ was synthesized using a facile hydrothermal treatment. Typically, 0.17 g of copper (II) chloride dihydrate (CuCl_2_·2H_2_O), 0.3 g of thioacetamide (TAA), and 0.29 g of indium (III) chloride tetrahydrate (InCl_3_·4H_2_O) were mixed in 20 mL of deionized water. Then, the mixture was transferred to an autoclave with a Teflon liner and heated at 120 °C for 6 h. The obtained mixture was centrifuged, dried at 70 °C, and labelled as CIS.

### 2.4. The Synthesis of xCIS/GCN

A total of 1.0 g GCN, 0.17 g of copper (II) chloride dihydrate (CuCl_2_·2H_2_O), 0.3 g of thioacetamide (TAA), and 0.29 g of indium (III) chloride tetrahydrate (InCl_3_·4H_2_O) were mixed in 50 mL deionized water. The dispersions were heated to 120 °C and calcined at 550 °C. As the amount of copper (II) chloride dihydrate, TAA, and indium (III) chloride tetrahydrate was different and proportional to the chemical formula, the synthesized sample was labelled 1CIS/GCN (0.17 g CuCl_2_·2H_2_O), 1.5CIS/GCN (0.255 g CuCl_2_·2H_2_O) 2CIS/GCN (0.34 g CuCl_2_·2H_2_O), 3CIS/GCN (0.51 g CuCl_2_·2H_2_O), and 4CIS/GCN (0.68 g CuCl_2_·2H_2_O).

### 2.5. Characterization of Catalysts

The characterization of the photocatalysts was carried out using X-ray powder diffraction (XRD, Smartlab SE, Tokyo, Japan), X-ray photoelectron spectroscopy (XPS, Nexsa G2. Thermo Fisher, Waltham, MA, USA), and Fourier-transform infrared spectroscopy (FT-IR, Nicolet iS5, Thermo Fisher, USA). The morphologies were investigated using scanning electron microscopy (SEM, Zep tools, Hefei, China) and transmission electron microscopy (TEM, JEM F200, Tokyo, Japan). The EPR test was performed to detect the reactive oxygen species on a Bruker X-band spectrometer with DMPO and TEMP as the spin-trapping agents.

### 2.6. Experimental Procedure

LVF was selected as the target pollutant to evaluate the catalytic performance of xCIS/GCN in the vis-xCIS/GCN/PMS process. The photocatalytic activity, stability, and recyclability were revealed through a series of batch experiments under different conditions, such as initial pH value, catalyst dosage, PMS concentration, and coexisting ion concentrations. A 300 W xenon lamp was used as the light source (Xe lamp, Microsolar 300, PerfectLight, Beijing, China). At the beginning of the experiment, 0.5 g L^−1^ xCIS/GCN was vigorously stirred for at least 1 h; then, 5 mM PMS was dispersed in the solution, and the Xe lamp was turned on. The samples were collected at different time intervals and filtered through a 0.22 μm membrane to analyze the concentration of LVF. The LVF concentration was measured using a visible-ultraviolet spectrophotometer (Nanjing Feile Instrument Co., Ltd., Nanjing, China), and the absorption peak intensity was at 289 nm. Other contaminants, such as RhB, Orange II, and phenol, were also detected by the UV–vis spectrometer to verify the universality of the catalyst. In order to evaluate the reusability of the catalyst, the catalyst was filtered and washed with ultrapure water, and then dried in an oven at 70 °C. The concentration of metallic ions in aqueous medium was determined using the ICP method (Agilent 720, Santa Clara, CA, USA). The degradation intermediates were identified using LC–MS/MS (Waters Q-tof, Milford, MA, USA), and a proposed degradation pathway was given. The radical quenching experiments were carried out using MeOH, TBA, and p-BQ to trap the sulfate radical, hydroxyl radical, and superoxide radical.

## 3. Results

### 3.1. Physicochemical Properties of the xCIS/GCN Heterojunction

**Figure 1:**
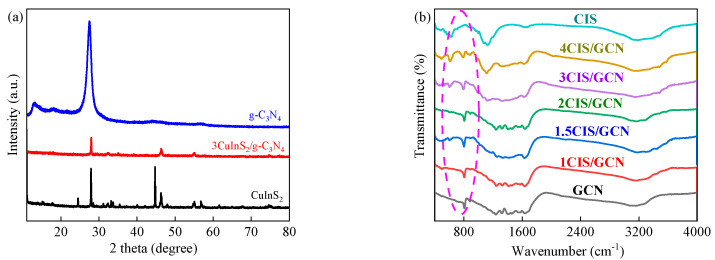
XRD patterns of CIS, GCN, and *3*CIS/GCN (**a**); FTIR spectra of the series *x*CIS/GCN (**b**).

The crystal structure of the series xCIS/GCN catalysts was analyzed using XRD characterization, as shown in Figure 1a and Figure S1. For the pure GCN sample, the intense diffraction peaks at 12.8° (100) and 27.7° (002) correspond to the interlayer stacking of aromatic structures and the in-plane ordering of tri-s-triazine systems [59]. Compared with the standard pattern of GCN (JCPDS: No. 87-1526), it is observed that no obvious impurity peaks or deviation occurred in the pattern. The main diffraction peaks of CIS are located at 27.9°, 32.4°, 44.6°, and 55.1°, which correspond to the (112), (200), (204), and (312) planes, respectively, in the tetragonal crystalline phase of CIS (JCPDS: No. 75-0106). Compared to pure CIS, several diffraction peaks in xCIS/GCN shifted to higher angles, indicating that the interaction between CIS and GCN is firming during the formation of the heterojunctions. The XRD patterns of 3CIS/GCN obtained less intensity compared to the pure GCN, which might be attributed to the strong interaction in the heterojunction between the CIS and GCN [60]. To explore the stability of the catalysts, the XRD pattern of 3CIS/GCN after the catalytic reaction was also investigated, as shown in Figure S2. No obvious changes were observed in the used catalyst.

To find the chemical structure and surface functional groups in the as-synthesized xCIS/GCN samples, Fourier-transform infrared (FTIR) spectra were used and are shown in Figure 1b. The absorption band at 806 cm^−1^ could be attributed to the stretching of the triazine ring structure in pure GCN, which is a typical characteristic peak of GCN. In the region of 1200–1600 cm^−1^, the vibration peaks are ascribed to the typical C–N/C=N vibration in the GCN structure [61]. The absorption bands in the region of 3000–3300 cm^−1^ can be assigned to the vibrations of O–H and N–H groups [62]. For the series xCIS/GCN samples, the FTIR spectra showed similar bands without obvious new bands, and the main typical characteristic peaks remained unchanged, proving that the participation of CIS in the GCN structure was successful without destroying the chemical structure of GCN.

**Figure 2:**
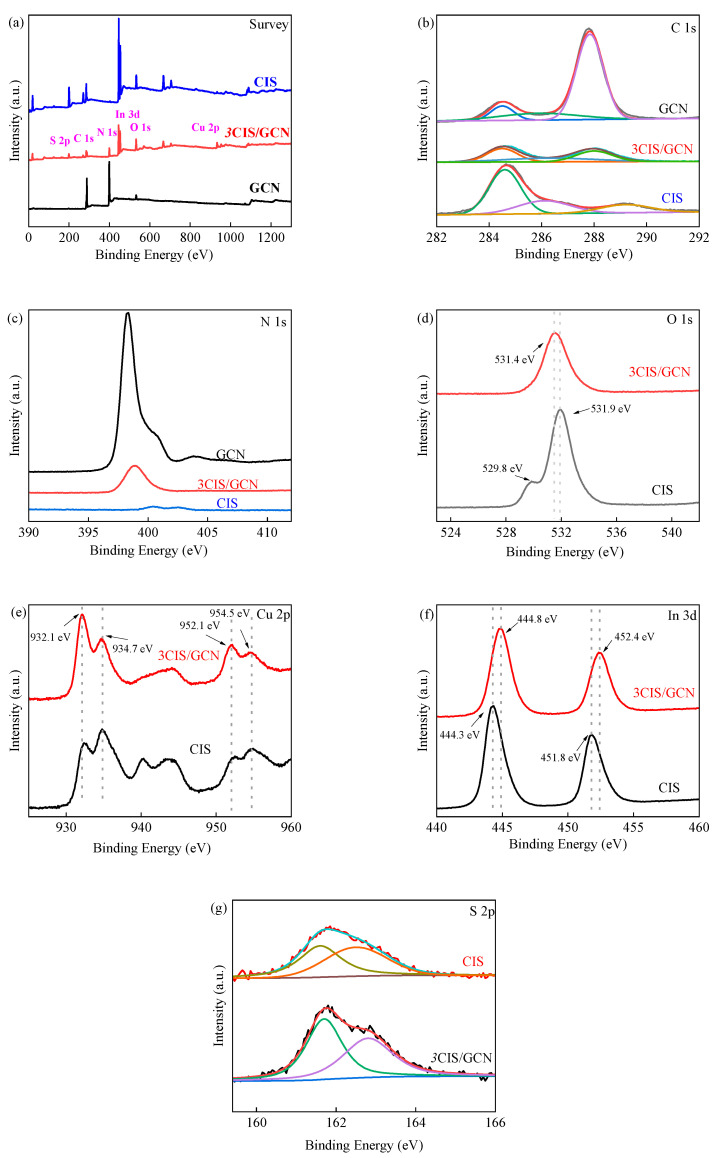
XPS survey spectra of CIS, GCN, and 3CIS/GCN (**a**); C 1s spectra (**b**); N 1s spectra (**c**); O 1s spectra (**d**); Cu 2p spectra (**e**); In 3d spectra (**f**); and S 2p spectra (**g**).

Moreover, the chemical composition and state of the series xCIS/GCN were investigated using X-ray photoelectron spectroscopy (XPS), as shown in Figure 2. The XPS survey spectra showed that C, N, and O elements were all present in the GCN, CIS, and 3CIS/GCN catalysts. At the same time, Cu, In, and S elements were present, which came from pure CIS and CIS embedding into the GCN structure. Three typical peaks of C 1 s were observed and located at 284.5 eV, 285.9 eV, and 287.8 eV, which were assigned to the N–C=N, C–NH_x_, and C atoms, respectively [62]. There are three peaks in the N 1 s high-resolution spectrum, located at 398.3 eV, 400.7 eV, and 404.1 eV, which are ascribed to the C–N=C, N–C_3_ and –NHx groups and π excitation, respectively [63]. In contrast to pure GCN, the binding energy in the 3CIS/GCN heterojunction shifted to a lower energy, which could be attributed to the electron transfer between the CIS and GCN during the heterojunction process [61]. In the high-resolution spectrum of O 1 s, peaks located at 531.4 eV and 531.9 eV were observed in 3CIS/GCN and CIS, respectively, which can be ascribed to the stretching of the O–H peak. In the high-resolution spectrum of S 2p, the peaks at 161.7 and 162.5 eV can be ascribed to S 2p_3/2_ and S 2p_1/2_, respectively. The S 2p spectra can be divided into two peaks, which proves that two chemical environments exist. In the Cu 2p high-resolution spectrum, the characteristic peaks at 932.1 eV and 952.1 eV are ascribed to Cu 2p_3/2_ and Cu 2p_1/2_ in the Cu (+1) species, and the satellite peaks at 934.7 eV and 954.5 eV are ascribed to Cu 2p_3/2_ and Cu 2p_1/2_ in the Cu (+2) species. The auxiliary peak of 944.0 eV is also observed in CIS, which is stronger than in the 3CIS/GCN samples. The results showed that the Cu species were present in the +1 state and +2 state. The XPS spectra of In 3d could be divided into two peaks located at 444.8 eV (In 3d_5/2_) and 452.4 eV (In 3d_3/2_), indicating that the In species was more present in the +3-oxidation state. The binding energies of Cu 2p, In 3d, and S 2p in the 3CIS/GCN spectra are slightly shifted to higher binding energies due to the strong interfacial interactions among these elements in the heterojunction composite and charge migration between CIS and GCN [64].

**Figure 3:**
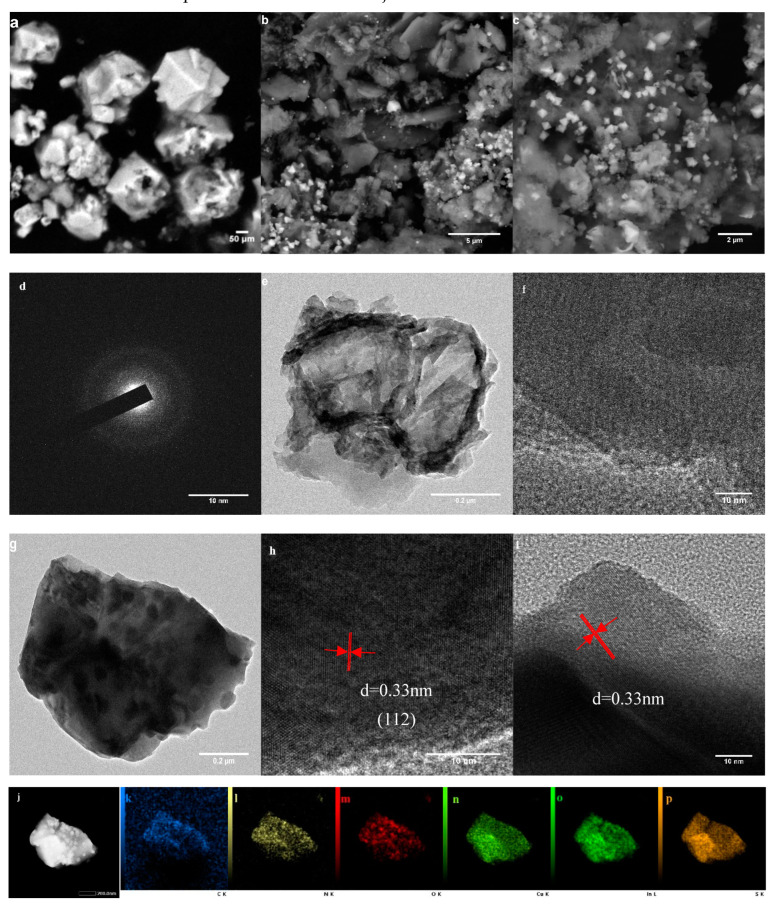
SEM images of CIS (**a**), 3CIS/GCN (**b**,**c**); TEM images of GCN (**d**–**f**); TEM images of 3CIS/GCN (**g**–**i**); and elemental distribution of C, N, O, Cu, In, and S (**j**–**p**).

The morphology of GCN, CIS, and xCIS/GCN was determined using scanning electron microscopy (SEM) and is shown in Figure 3a–c. Compared to the CIS samples, the GCN sample exhibits a thin lamellar morphology. The CIS particles are observed to have an octahedral structure and aggregate together as a spherical bulk. The 3CIS/GCN catalyst also showed a thin lamellar morphology, and the CIS particles were embedded into the multi-layered structure, which provided an adequate platform for the sequenced photocatalytic reaction. Transmission electron microscopy (TEM) images of GCN (Figure 3d–f) and 3CIS/GCN (Figure 3g–i) are displayed. It is observed that the CIS particles were uniformly dispersed on the surface of the GCN nanosheets with a lattice fringe of 0.33 nm. The interface boundary between CIS and GCN can be clearly observed, indicating that the interaction between CIS and GCN was strong, which takes great advantage of continuous photocatalytic degradation. The multiple sheet-like structure in the 3CIS/GCN catalyst could provide more active sites on the platform and more absorption sites, facilitating the sequence of the photocatalytic process. The EDS surface scanning method was utilized to verify the corresponding elemental mappings of C, N, O, Cu, In, and S at the interface and is displayed in Figure 3j–p, proving the lamellar morphology in the 3CIS/GCN catalyst. In the spectrum, the elements C, N, O, Cu, In, and S are observed at the interface, confirming the presence of a close heterojunction between CIS and GCN in the 3CIS/GCN catalyst.

**Figure 4:**
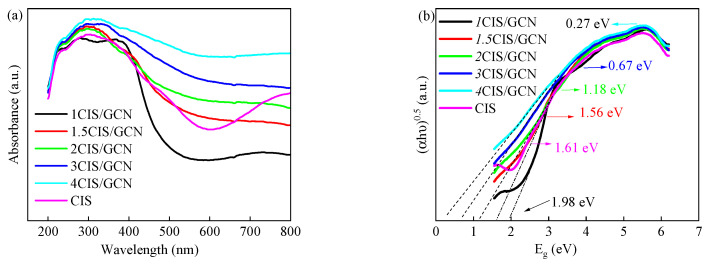
CIS and xCIS/GCN: (**a**) UV–vis diffuse reflectance spectra; (**b**) band gap energy spectra.

The optical properties of the xCIS/GCN samples were investigated using UV–vis adsorption spectra, as shown in Figure 4. The absorption spectra showed that the absorption edge of synthesized xCIS/GCN was approximately in the range of 200–440 nm, which is in good agreement with the results of pure GCN [65]. The absorption intensity was enhanced in the series catalyst of xCIS/GCN, while the visible range was increased with the CIS loading composites. Moreover, the band gap energy (E_g_) of catalysts can be calculated by the Kubelka–Munk function, which was calculated to be 1.98 eV (1CIS/GCN), 1.56 eV (1.5CIS/GCN), 1.18 eV (2CIS/GCN), 0.67 eV (3CIS/GCN), 0.27 eV (4CIS/GCN), and 1.61 eV (CIS). The results demonstrated that the light response range was prolonged and the ability to trap visible light was promoted in these xCIS/GCN catalysts rather than pure catalysts, which facilitated the promotion of more photogenerated pairs and enhanced photocatalytic activity.

### 3.2. Catalytic Performance Evaluation of CIS/GCN

**Figure 5:**
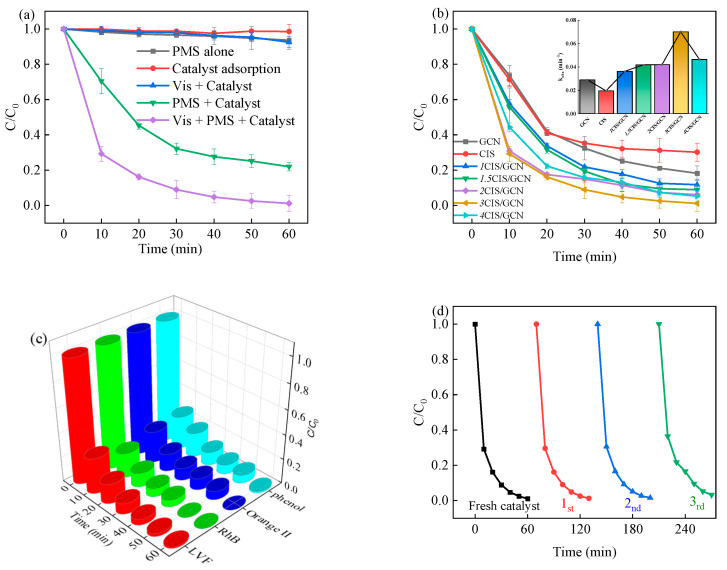
Photocatalytic degradation of LVF in different systems (**a**); with xCIS/GCN as the catalyst in the visible-light-driven PMS activation (**b**); the degradation performance of different pollutants over 3CIS/GCN catalyst (**c**); and reusability tests of the 3CIS/GCN catalyst (**d**).

To evaluate the catalytic performance of synthesized xCIS/GCN heterojunctions in the activation of PMS under visible light irradiation for the elimination of levofloxacin (LVF), batch experiments were carried out. The adsorption efficiency of 3CIS/GCN was investigated in the absence of PMS and visible light irradiation. As shown in Figure 5, the absorption equilibrium was reached within 60 min and the absorption efficiency was less than 10% of LVF removal. With the addition of PMS, the LVF degradation efficiency increased to 78.1% as the sulfate radicals were generated during the process. However, by applying PMS alone in the degradation process, the degradation efficiency was 6.4%, where the degradation of LVF occurred through the self-decomposition of PMS at room temperature. Both experiments revealed that the PMS self-decomposition and catalyst absorption have little degradation efficiency to the LVF degradation. The photocatalysis process also showed a poor removal efficiency of LVF, which was 7.5%, due to the high combination efficiency of photogenerated electrons and holes, leading to an unsatisfactory removal rate. With the addition of PMS in the photocatalysis system, the LVF degradation efficiency was significantly promoted and increased to 98.9% in 60 min. The degree of synergy between the systems was quantified to 62.1% by using 3CIS/GCN materials in the vis-PMS activation process according to Equation (S1) [66]. Synergistic effect was found in the process with a high degree of synergy. The impact of CIS loading on the degradation efficiency was also investigated and is shown in Figure 5b. It is obvious that the 3CIS/GCN/vis-PMS system performs best among other catalysts, while the removal efficiency is 88.3%, 91.1%, 93.9%, 94.8%, 81.8%, and 69.8% in that of 1CIS/GCN, 1.5CIS/GCN, 2CIS/GCN, 4CIS/GCN, pure GCN, and CIS, respectively. The results showed that the heterojunctions can facilitate the visible-light-driven PMS activation process due to the different photosensitivity of the catalysts. The removal efficiency of LVF first increased and then decreased with the loading of CIS, which may be probably due to bulk CIS aggregation on the surface of GCN, which was confirmed by the SEM and TEM images. The excess CIS loading has a negative effect on the contact between active sites and photogenerated pairs, leading to decreased degradation efficiency. In order to investigate the universality of the 3CIS/GCN catalysts, Orange II, rhodamine B, and phenol were selected as target pollutants, as shown in Figure 5c. The degradation efficiency was 100%, 100%, and 98.7% in 60 min in the 3CIS/GCN/vis-PMS process, respectively. The results confirmed the excellent catalytic activity of the various compounds with low PMS concentrations and catalyst dosages. Furthermore, the mineralization of LVF in the 3CIS/GCN/vis-PMS process was satisfactory, which was 61.5% after 60 min of reaction. In addition, the synthesized xCIS/GCN materials were compared with other similar C_3_N_4_-based (CN-based) materials which have been published earlier. Comparing this work with previous reports, the prepared composite catalyst also showed high catalytic activity, as shown in Table S1.

**Figure 6:**
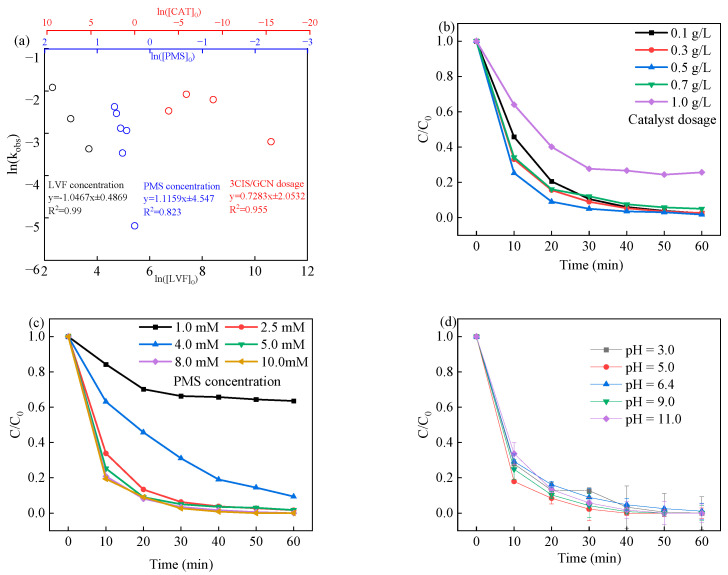
The linear correlation between ln (k_obs_) and ln (concentration) in the kinetic study (**a**); the effects of catalyst dosage (**b**), PMS concentration (**c**), and pH value (**d**) on LVF degradation.

The control experiments to verify the various reaction conditions for the removal of LVF over 3CIS/GCN in visible-light-driven PMS activation were further explored, including catalyst dosage, PMS concentration, initial solution pH, coexisting ions, and natural organic matter (NOM). To explore the effect of catalyst dosage on PMS activation, different amounts of 3CIS/GCN were employed. As shown in Figure 6b, the LVF removal efficiency increased from 97.5% to 98.9% with increasing catalyst dosage, and the k_obs_ value of LVF degradation increased from 0.059 min^−1^ to 0.0701 min^−1^. As the catalyst dosages increased from 0.1 to 0.5 g L^−1^, more photocatalytic reaction sites were present with the contact of PMS, leading to more production of reactive species. When the catalyst dosage reached close to 1.0 g L^−1^, although the degradation efficiency gradually increased to 74.4%, the reaction rate reached 0.0229 min^−1^. The excess catalyst in the system can provoke a quenching reaction of reactive species with each other [67]. A similar trend can be obtained in the effect of PMS concentration, while the increase in PMS concentration boosted the removal efficiency of LVF, as shown in Figure 6c. The kinetic constants in the system increased from 0.007 min^−1^ to 0.0701 min^−1^ as the PMS concentration ranged from 1 to 5 mM. The increase in PMS concentration in the system can produce more reactive species, especially sulfate radicals, leading to the improvement of removal efficiency. However, the excess of PMS concentration can arouse the occurrence of radical quenching experiments, leading to decreased removal efficiency and rate [68]. The linear relationships between the ln (effect parameters) (effect parameters were 3CIS/GCN dosage, LVF concentration, and PMS concentration) and ln (k_obs_) were studied to explore the key parameters during the visible-light-driven PMS activation process, as shown in Figure 6a. The corresponding slopes of ln (3CIS/GCN), ln (LVF concentration), and ln (PMS concentration) were calculated to be 0.7283, 1.0467, and 1.1159, respectively. It is recognized that the higher the corresponding slopes are, the more significant the influence of the effect parameters is [1]. In this case, PMS concentration is the key effect parameter in the visible-light-driven PMS activation system. The effect of the initial pH value on LVF degradation in PMS activation assisted by the visible light system was investigated and is shown in Figure 6d. The initial pH of the reaction solution was close to 6.0, while the LVF was nearly completely removed in 60 min. When the pH was up to 9.0 and 11.0, it is observed that the LVF removal efficiency was enhanced due to the activation of PMS under alkali conditions. On the other hand, the degradation efficiency was slightly decreased at pH 3.0, which can be ascribed to the quenching experiment of free radicals, leading to a decrease in the number of radicals [69]. Nevertheless, the LVF degradation efficiency remained high over a wide range of initial pH values from 3.0 to 11.0, indicating the good practical application of the catalyst in wastewater treatment.

**Figure 7:**
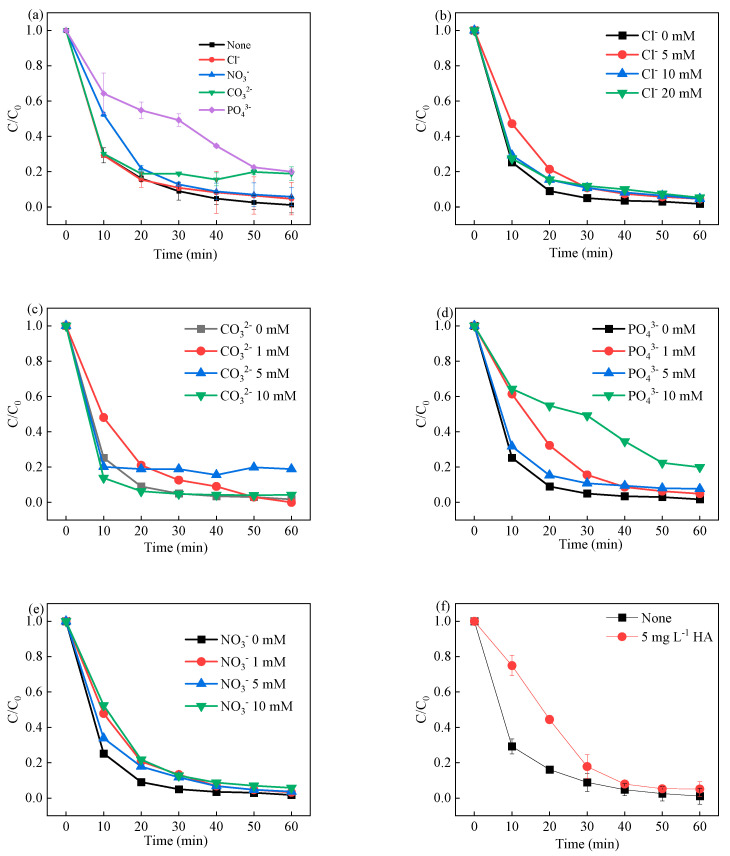
Influence of common anions (**a**), Cl^−^ concentration (**b**), CO_3_^2−^ concentration (**c**), PO_4_^3−^ concentration (**d**), NO_3_^−^ concentration (**e**), and humic acid (**f**) on LVF degradation in the 3CIS/GCN/vis-PMS system.

The coexisting anions can greatly affect the refractory chemical degradation efficiency in the system (Figure 7a). In the presence of Cl^−^, the LVF degradation efficiency was slightly decreased, as shown in Figure 7b. It is considered that the PMS and reactive species could react with Cl^−^, leading to the formation of less active oxidizing chlorine species, as shown in Equations (1)–(3). As shown in Figure 7c, the LVF removal efficiency was decreased by the presence of CO_3_^2−^, which is considered a quenching agent for the sulfate radical and hydroxyl radical (Equations (4) and (5)). A similar trend of decreased removal efficiency was also found in the presence of PO_4_^3−^. In the presence of PO_4_^3−^ (Figure 7d), the removal efficiency decreased to 80.7% and 94.2%, respectively. It is considered that the PO_4_^3−^ ions can scavenge the sulfate radical and hydroxyl radicals, leading to the generation of less reactive species. The slight inhibition of NO_3_^−^ ions on LVF degradation implied that the 3CIS/GCN/Vis-PMS system is highly tolerant to NO_3_^−^ ions (Figure 7e). The effect of coexisting ions followed the order: PO_4_^3−^ > CO_3_^2−^ > Cl^−^ ≈ NO_3_^−^. It is known that natural organic matter (NOM) has a great impact on the degradation efficiency of these chemical compounds, which can react with PMS, sulfate radicals, and hydroxyl radicals, leading to the decreased degradation efficiency of the chemical compounds. Humic acid (HA) was chosen to investigate the effect of NOM in the 3CIS/GCN/vis-PMS process. As shown in Figure 7f, the LVF degradation efficiency was inhibited in the presence of HA due to the scavenging effect of NOM on free radicals. In conclusion, the 3CIS/GCN catalyst is successfully applied in the visible-light-driven PMS activation process for the degradation of LVF, exhibiting good catalytic performance for pollutant removal.

SO_4_^•−^ + Cl^−^ → SO_4_^2−^ + Cl^•−^, (1)

^•^OH + Cl^−^ → ClOH^•−^, (2)

ClOH^•−^ + H^+^ → Cl^•^ + H_2_O, (3)

CO_3_^2−^ + H_2_O → HCO_3_^−^ + OH^−^, (4)

SO_4_^•−^ + HCO_3_^−^ → SO_4_^2−^ + HCO_3_^•^, (5)

^•^OH + HCO_3_^−^ → H_2_O + CO_3_^•−^, (6)

SO_4_^•−^ + NO_3_^−^ → SO_4_^2−^ + NO_3_^•^, (7)

### 3.3. Identification of Reactive Species and Mechanism in the Process

**Figure 8:**
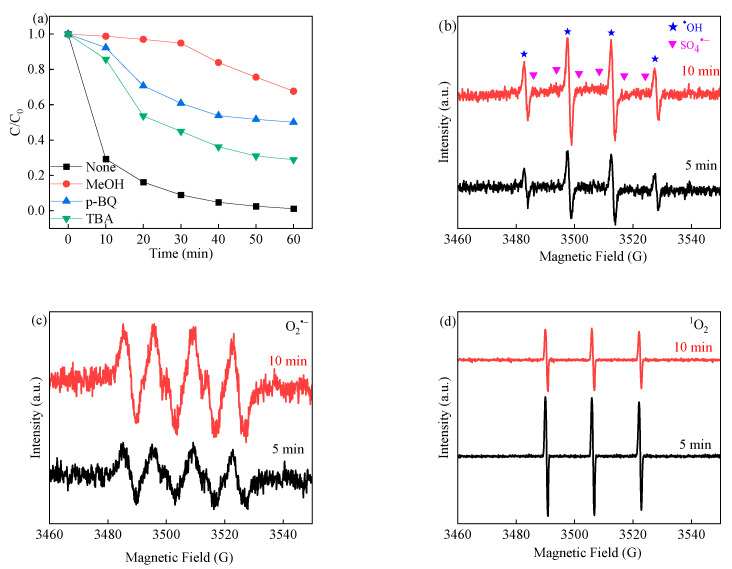
Effects of scavengers on LVF degradation in the 3CIS/GCN/vis-PMS/ system (**a**); ESR trapping signals of DMPO-SO_4_^•−^ and DMPO-^•^OH (**b**), DMPO-O_2_^•−^ (**c**), and TEMP-^1^O_2_ (**d**).

Free radicals play a key role in the visible-light-assisted PMS activation process as the active species. In this case, trapping experiments were carried out to investigate the contribution of these reactive species, as shown in Figure 8a. It is known that methanol (MeOH) and tert-butyl alcohol (TBA) are usually used as scavengers in the SR-AOP system. MeOH is considered a scavenger for sulfate radicals (1.6–7.7 × 10^7^ M^−1^ s^−1^) and hydroxyl radicals (1.2–1.8 × 10^9^ M^−1^ s^−1^), while TBA is the main scavenger for hydroxyl radicals (3.8–7.6 × 10^8^ M^−1^ s^−1^) rather than sulfate radicals (4.0–9.1 × 10^5^ M^−1^ s^−1^) [70]. Then, p–benzoquinone (p–BQ) is considered a trapping agent for O_2_^•−^ (0.9–1.0 × 10^9^ M^−1^ s^−1^) [71]. The degradation efficiency was slightly inhibited to 49.9% when p–BQ was set at 50 mM. Moreover, in the presence of MeOH, the LVF degradation efficiency was greatly influenced and decreased to 32.4% in 60 min of reaction time. The results demonstrated that sulfate radicals were produced during the reaction and played a substantial role in LVF degradation. After the addition of 50 mM TBA, the degradation efficiency slightly decreased to 71.1%, indicating that hydroxyl radicals appeared in the system and had less impact than sulfate radicals in the system. All of the reactive species can be present in the system but offer different degrees of impact. Therefore, sulfate radicals are superior to hydroxyl radicals and superoxide radicals in terms of their influence on the LVF removal efficiency.

To verify the above conclusions, EPR experiments were conducted by using DMPO and TEMP as the spin-trapping agents. As the reaction began, the characteristic peak signals of hydroxyl radicals appeared (1:2:2:1), implying the presence of hydroxyl radicals in the vis-SR-AOP system. The DMPO-SO_4_^•−^ peak signals (1:1:1:1:1:1) were also observed, and the signals were weak, which might be ascribed to the transition of sulfate radicals to hydroxyl radicals through Equation (8). Notably, the intensity of the EPR signals belonging to TEMP-^1^O_2_ was observed, which showed a 1:1:1 pattern and increased with the reaction time, implying the participation of singlet oxygen in the 3CIS/GCN/vis-PMS system. Therefore, the TEMP-^1^O_2_ signal might be produced due to the contribution of superoxide in the system (Figure 8d). As shown in Figure 8c, the EPR signals of DMPO-O_2_^•−^ were also detected in the process, and the signal intensity increased with the reaction time. Based on the above results, all of the sulfate radicals, hydroxyl radicals, superoxide radicals, and singlet oxygen were detected and considered as the reactive species in the visible-light-assisted PMS activation system.

SO_4_^•−^ + OH^−^ → SO_4_^2−^ + ^•^OH, (8)

SO_4_^•−^ + H_2_O → ^•^OH + SO_4_^2−^ + H^+^, (9)

Cu^2+^ + HSO_5_^−^ → Cu^+^ + SO_5_^•−^ + H^+^, (10)

SO_5_^•−^ + H_2_O → 2HSO_4_^−^ + 1.5 ^1^O_2_, (11)

HSO_5_^−^ → SO_4_^2−^ + H^+^ + 0.5 ^1^O_2_, (12)

O_2_ + e^−^ → O_2_^•−^, (13)

O_2_^•−^ + H_2_O → ^•^H_2_O_2_, (14)

h^+^ + H_2_O → ^•^OH + H^+^, (15)

**Figure 9:**
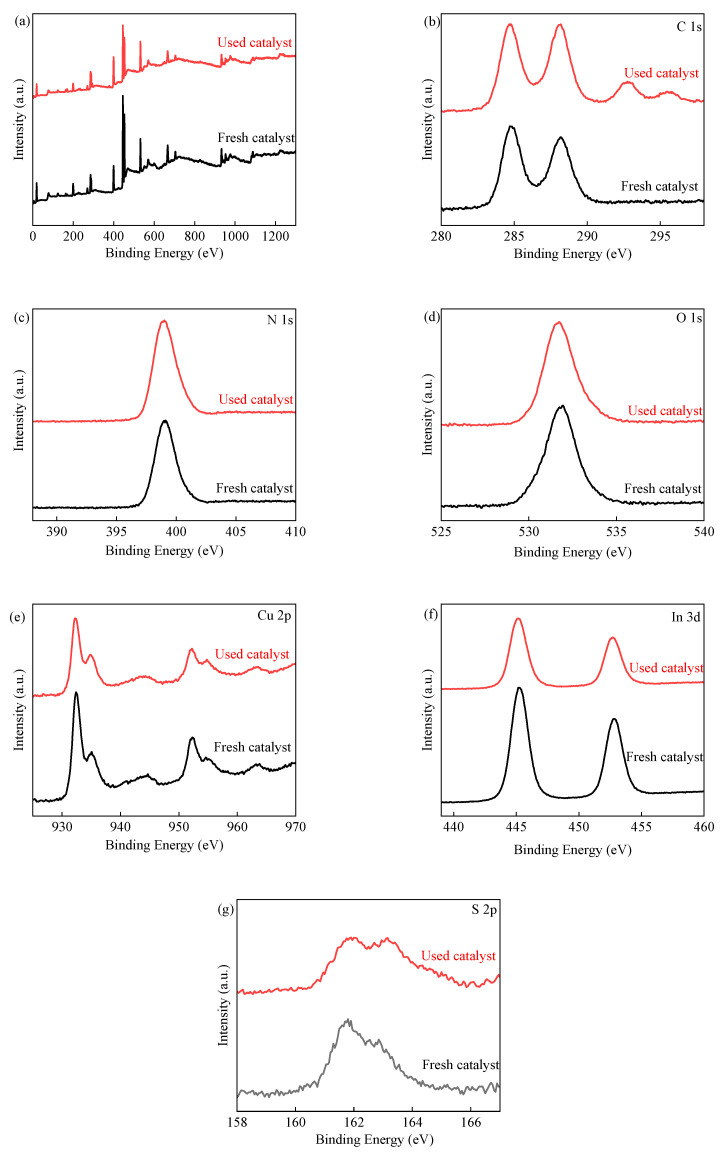
XPS spectra of fresh and used 3CIS/GCN catalyst, XPS survey (**a**); C 1s spectra (**b**); N 1s spectra (**c**); O 1s spectra (**d**); Cu 2p spectra (**e**); In 3d spectra (**f**); and S 2p spectra (**g**).

To explore the mechanism of reactive species in the system, XPS spectra were employed to identify the difference in the chemical state and composition of surface elements between fresh and used 3CIS/GCN catalysts, as shown in Figure 9. No obvious spectral peak shifts were obtained after 3CIS/GCN was used for visible-light-driven PMS activation. The Cu^+^/Cu^2+^ ratio increased after the reaction, indicating the participation of electrons in the reaction. The proportion of lattice oxygen decreased, while the proportion of absorbed oxygen increased. It is proven that the reaction concludes the reaction of metal redox and the formation of surface hydroxyl groups in the system. The oxygen vacancy was involved in the reaction as the proportion of lattice oxygen decreased, which can accelerate the metal redox reaction as well as the photocatalytic reaction. For the S 2p peak region, after the reaction, the S 2p_1/2_ peak shifted to a higher binding energy region due to the presence of SO_4_^2−^, which can be formed by the decomposition of PMS and the oxidation of S^2−^. In the peak of In 2p, the peak slightly shifted to a higher binding energy region, while the curve of the peak decreased in contrast to the fresh catalyst. The results suggest that the In species also participate in the reaction.

**Scheme 1:**
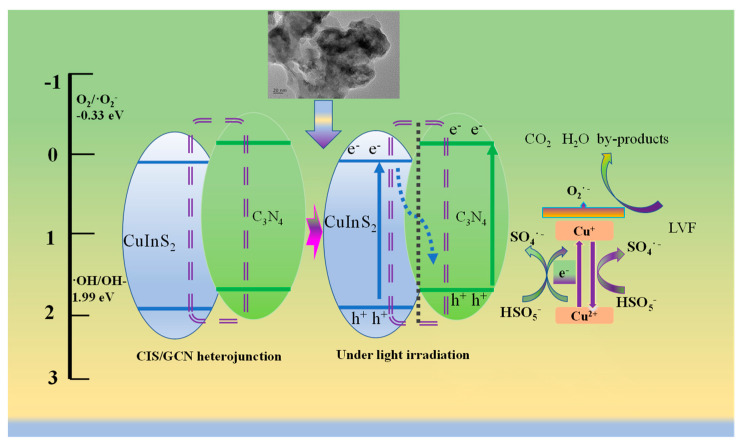
The mechanism of 3CIS/GCN photo-assisted activation of PMS for LVF degradation.

Based on the above results, a mechanism was proposed and is shown in Scheme 1. The heterojunction of 3CIS/GCN catalyst can absorb visible light, while photogenerated electrons appear in the corresponding CBs and photogenerated holes in the VBs. The excited electrons will combine with the photogenerated holes and react with dissolved oxygen to produce superoxide species for LVF degradation. The photogenerated holes can also directly react with water and PMS, and then hydroxyl radicals and SO_4_^•−^ are formed. Hydroxyl radicals can also be produced through reactions with sulfate radicals and hydroxyl groups/water. Meanwhile, the photogenerated electrons can easily transfer to metal active species with the assistance of a carbon layer, while superoxide can also be generated through the reaction between the electrons and absorbed oxygen on the surface of the catalyst [13]. Then, the formation process of singlet ^1^O_2_ can be reasonably speculated that the reaction between Cu and In species with PMS could generate SO_5_^−^, and singlet oxygen (^1^O_2_) can be produced due to the reaction with water. On the other hand, the self-decomposition of PMS can also generate singlet ^1^O_2_. The heterojunctions formed between CIS and GCN with different conduction bands (CBs) and valence bands (VBs) can efficiently prevent the recombination of photogenerated pairs, leading to better catalytic efficiency. Moreover, the redox cycle of different valences in metal species can be accelerated by the photogenerated pairs with the assistance of a carbon layer in GCN. Visible light irradiation can bring electrons into the solution, leading to the production of superoxide and singlet oxygen (^1^O_2_), which participate in the reaction, leading to the good removal efficiency of LVF in the heterogeneous PMS activation process. Subsequently, the reactive oxygen species in the solution plays a positive role in the degradation of LVF in the visible-light-driven PMS system, which verifies the synergistic effect of photocatalysis and the heterogeneous PMS activation process.

### 3.4. Degradation Pathway of LVF

**Figure 10:**
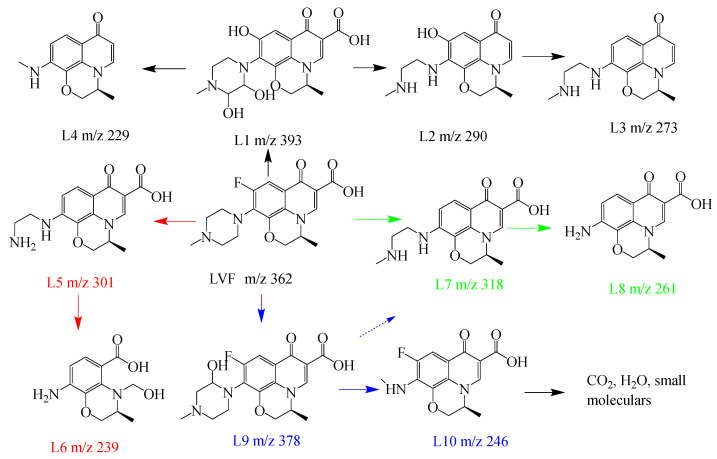
Possible LVF degradation pathways over 3CIS/GCN in the visible-light-driven PMS activation process.

The by-products of LVF degradation were identified using liquid chromatography–mass spectrometry (LC–MS) in the 3CIS/GCN/vis-PMS process. The total MS spectra of the by-products are displayed in Figure S5, and the possible structures of the by-products are shown in Table S2. The corresponding investigations on LVF degradation by a similar system, which was the visible-light-driven PMS activation process, are shown in Table S3. Based on the possible by-products, four degradation pathways can be proposed and are shown in Figure 10. The molecular weight of LVF in the ion spectra is at *m*/*z* = 362. In pathway I, product L1 at *m*/*z* = 393 can be considered as the replacement of F by the hydroxyl groups in the solution, which is further degraded to products L2 (*m*/*z* = 290) and L3 (*m*/*z* = 273) through the decarboxylation and dihydroxylation process. In pathway II, the fluorine was removed, and the piperazine ring was destroyed, leading to the formation of L7. The cleavage of piperazinyl groups led to the transformation of L8. In pathway III, the cleavage of the C-N bond and the shedding of fluorine groups in the LVF structure led to the occurrence of L5. Then, L6 was formed due to the sequential oxidation of the C-C bond and hydroxylation process. In pathway IV, L9 was formed as a hydroxylation product, which was further degraded to product L10 through the destruction of the piperazine ring and decarboxylation due to the reaction with reactive species, such as sulfate radicals, hydroxyl radicals, and/or reactive oxygen species [72]. Finally, these by-products were degraded to small molecules such as CO_2_ and H_2_O in the 3CIS/GCN/vis-PMS system.

### 3.5. Reusability and Stability

The reusability of the synthesized catalyst is a great factor in judging the actual usefulness of the catalyst. To investigate the reusability of 3CIS/GCN in the visible-light-driven PMS activation process, cycle experiments for LVF degradation were carried out, as shown in Figure 5d. In the three recycling experiments, the LVF degradation efficiency still remained high. Although the degradation efficiency was gradually decreased in the cycle experiments, the degradation efficiency was still maintained in contrast to that of the fresh catalyst. The decreased degradation efficiency might be attributed to the occupied active sites on the surface during the degradation process. In order to investigate the LVF removal efficiency in the real water matrix, different kinds of water were investigated. The results showed that the varying removal efficiency of tap water (47.7%), lake water (28.9%), and mineral water (66.5%) can be attributed to the inorganic ions, color, dissolved organic substance, and natural organic matters in these water matrices.

The XRD patterns of 3CIS/GCN before and after the reaction are shown in Figure S2. No obvious change was observed between the fresh and used catalysts of 3CIS/GCN, indicating that the structure was maintained during the degradation process without change. The FTIR spectra and SEM image of 3CIS/GCN used after the photocatalysis process are shown in Figures S3 and S4. For the used catalyst, the main absorption bands showed no obvious change compared to the fresh catalyst. The newly formed bands at 2920 and 2850 cm^−1^ can be ascribed to the stretching vibrations of CH_2_ groups, which are caused by the absorbed degradation intermediate of LVF. The functional groups of absorbed OH/H_2_O were stretching up at 1390 cm^−1^. The SEM images showed that the main structure was maintained after the reaction. Moreover, the leaching ions during the reaction were also investigated using ICP. The metal leaching concentration of 3CIS/GCN after the degradation process is still low, approximately 0.52 mg L^−1^ of Cu and 0.13 mg L^−1^ of In. The percentage of leached metal ions is approximately 2.67% and 3.80% in the applied atomic mass of 3CIS/GCN, which only obtained a small fraction of the catalyst dosage. The results proved that 3CIS/GCN is an efficient and durable catalyst in the visible-light-driven PMS activation process.

## 4. Conclusions

A series of xCIS/GCN materials were successfully prepared as efficient photocatalysts for the degradation of LVF, which were combined with CIS and pure GCN. With the composite materials, the photocatalytic oxidation of LVF significantly increased. The degradation rate of LVF solution with 3CIS/GCN was 98.9% in 60 min, which was much higher than that of pure g-C_3_N_4_. The optimized 3CIS/GCN exhibits the best catalytic efficiency for the removal of LVF. The catalyst possessed good reusability and stability in the process and can be employed with a wide pH range and anti-anion interference. The high photocatalytic efficiency of CIS/GCN was mainly attributed to the interaction of heterojunctions between CIS and GCN, which accelerated the separation of photogenerated pairs. It is an effort to amend the heterojunction of CIS with GCN and obtain a fresh idea for augmenting the performance of GCN. This work provides a feasible strategy for combining photocatalysis and heterogeneous PMS activation processes where a synergistic effect was found. The synthesized xCIS/GCN might be considered a promising candidate with good potential as an actual practice catalyst for refractory chemical compounds in wastewater treatment.

## Data Availability

Data are contained within the article.

## Supplementary Materials

The following supporting information can be downloaded at: https://www.mdpi.com/article/10.3390/nano14010074/s1. References [73,74,75,76,77,78,79,80,81,82,83,84,85,86,87] are cited in the supplementary materials.
