# Supplementary material for: Enhanced Degradation of Levofloxacin through Visible-Light-Driven Peroxymonosulfate Activation over CuInS2/g-C3N4 Heterojunctions
Source: Nanomaterials (Basel). 2023 Dec 26;14(1):74. doi: 10.3390/nano14010074 (PMC10781168; doi:10.3390/nano14010074)
Supplement: Supplementary file 1 [file nanomaterials-14-00074-s001.zip › nanomaterials-2776859-supplementary.pdf]

Supporting Information

Enhanced Degradation of Levofloxacin through Visible-Light-Driven Peroxymonosulfate Activation over $CuInS_2$/g-C_3N_4 Heterojunctions

Xin Zhong [1,2,*], Meihuan Ji [1], Wenxin Wu [1], Caicai Lu [1], Wenping Liu [1] and Fubin Jiang [1,*]

1 Experimental and Practical Innovation Education Center, Beijing Normal University at Zhuhai, Zhuhai 519087, China; meihuanji@bnu.edu.cn (M.J.); wxw1018@outlook.com (W.W.); lucaicai@bnu.edu.cn (C.L.); liuwp@bnu.edu.cn (W.L.)
2 Department of Environmental Engineering and Science, Beijing Normal University at Zhuhai, Zhuhai 519087, China
* Correspondence: zhongxin@bnu.edu.cn (X.Z.); jfb@bnu.edu.cn (F.J.)

Equations

The degree of synergy, S, was quantified according to the following equation:

$$S(\%) = \frac{k_{combined} - \sum_k^n ki}{k_{combined}} \times 100\% \qquad (S1)$$

S%: > 0, synergistic effect; = 0, cumulative effect; < 0, antagonistic effect

Figures

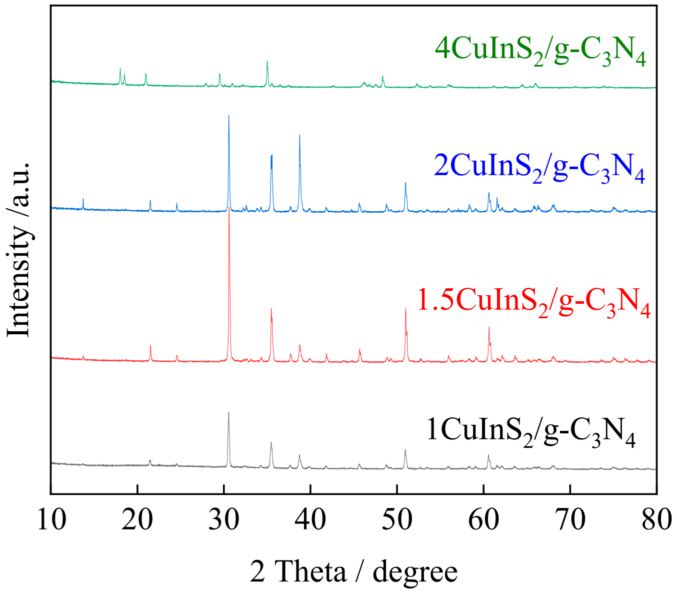

Figure S1 XRD patterns of series of *x*CIS/GCN catalysts.

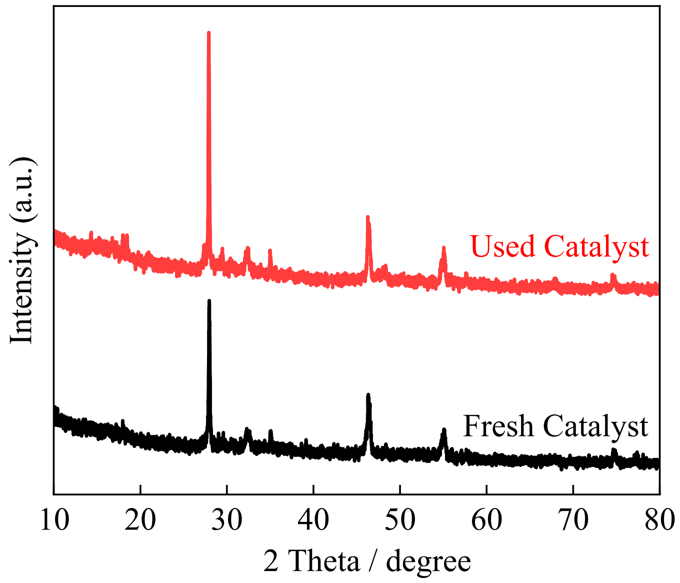

Figure S2 XRD patterns of fresh catalyst and used catalyst

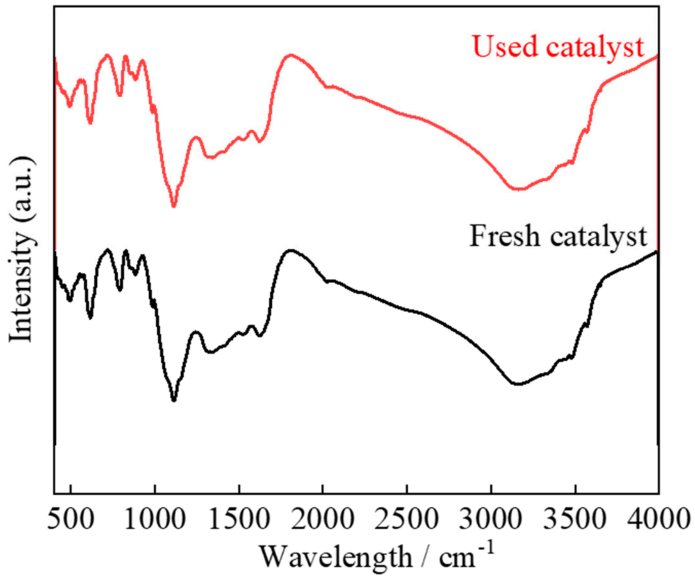

Figure S3 The FTIR spectra of fresh and used *3*CIS/GCN after the photocatalysisprocess.

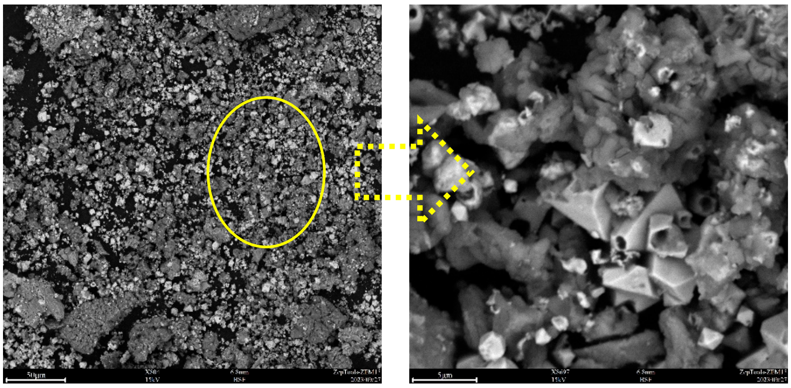

Figure S4 The SEM image of 3CIS/GCN used after the photocatalysis process.

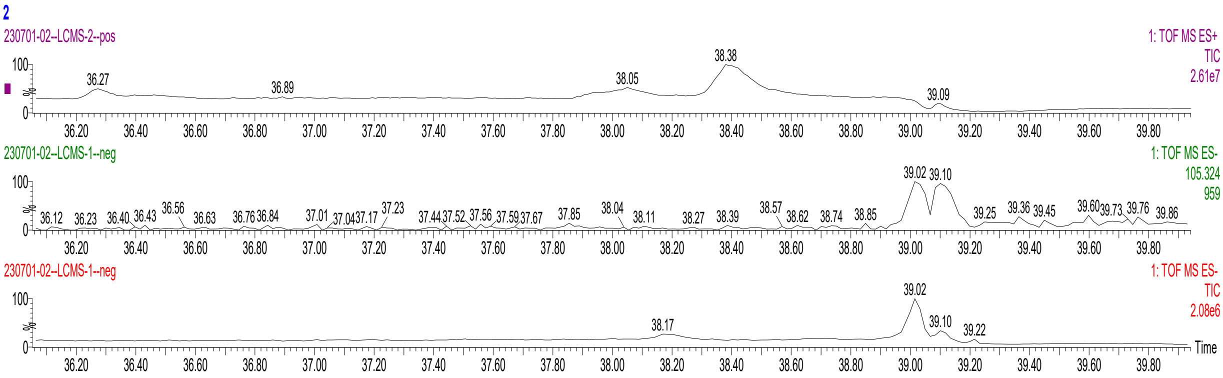

Figure S5 The total MS spectra of the byproducts of LVF.

Table S1 Studies on of various contaminant using Metal based C_3N_4 catalysts

No.	Systems	Catalyst	Target pollutant	Time/ efficiency	Operation parameters
1	Vis +PMS+ 1%Cu - GCN	copper single atom incorporated in C_3N_4	bisphenol A	25 min; 100%	PMS concentration: 40 mg/L; catalyst dosage: 0.33 g/L; BPA concentration: 20 mg/L; temperature: 25 ◦C
2	Vis+ PMS+ 5Cu-OCN	Cu single atoms/O doping g-C_3N_4	Carbamazepine (CBZ)	35 min; 97.8%	Catalyst: 50 mg; CBZ concentration: 10 μM; PMS concentration: 2 mM; pH: 9.0
3	Vis+10CIS/CN	$CuInS_2$ quantum-dot-modified g-C_3N_4 S-scheme heterojunction	tetracycline (TC)	120 min; 52.16%	TC concentration: 40 mg/L; Catalyst dosage: 0.5 g/L;
4	Vis+PMS +5Cu/CN	CuO/g-C_3N_4 2D/2D heterojunction photocatalysts	oxytetracycline (OTC)	20 min; 100%	pH = 5, T = 20 ◦C, [OTC] = 30 mg/L, [PMS] = 0.65 mM, [catalyst] = 0.2 g/L)
5	Sunlight+ PMS+Cu^+	Cu^+-decorated g-C_3N_4	RhB	30 min; 95.7%	RhB = 10 mg/L, catalyst dose = 0.5 g/L, PMS concentration

	/g-C_3N_4				= 0.5 mmol/L, initial pH = 7.09
6	Vis+10%g-C_3N_4/Cu-MOF	g-C_3N_4/Cu-MOF nanocomposite	microcystin	6h; 40.3%	Catalyst dosage: 10 mg/L; MC concentration: 50 μg/L;
7	Vis+50% CIS-CN	2D/2D Z-scheme heterojunction of $CuInS_2$/g-C_3N_4	Tetracycline (TC)	60 min; 83.7%	Catalyst dosage: 0.5 g/L; TC concentration: 10 mg/L
8	Vis+TiO_2/$CuInS_2$/OCN/CQD	Z-scheme photocatalyst TiO_2/$CuInS_2$/OCN incorporated with carbon quantum dots (CQDs)	reactive yellow 145 dye	60 min; 95.44%	Catalyst dosage: 0.3 g/L; [dye] concentration: 50 mg/L;
Our work	Vis+PMS +3CIS/GCN (this work)	$CuInS_2$/g-C_3N_4 heterojunctions	Levofloxacin (LVF)	60 min 98.7%	Catalyst dosage: 0.5 g/L PMS concentration: 5 mM LVF: 25 mg/L

[1] W. Huang, H. Ming, X. Bian, C. Yang, Y. Hou, K. Ding, J. Zhang. Copper single atoms incorporated in crystalline carbon nitride for efficient photocatalytic activation of peroxymonosulfate toward bisphenol A removal with visible light. Chemical Engineering Journal 473 (2023) 145230.

[2] Y. Xu, M. Guo, C. Ge, P. Zhang, W. Xu, L. Zhang, S. Zhou, J. Liao. Cu single atoms/O doping g-C_3N_4 mediated photocatalytic activation of peroxymonosulfate for ultrafast carbamazepine removal via high $^1O_2$ yield. Applied Surface Science 640 (2023) 158290.

[3] J. Zhang, Y. Zhao, K. Qi, S. Liu. $CuInS_2$ quantum-dot-modified g-C_3N_4 S-scheme heterojunction photocatalyst for hydrogen production and tetracycline degradation. Journal of Materials Science & Technology 172 (2024) 145–155.

[4] M. Wang, C. Jin, J. Kang, J. Liu, Y. Tang, Z. Li, S. Li. CuO/g-C_3N_4 2D/2D heterojunction photocatalysts as efficient peroxymonosulfate activators under visible light for oxytetracycline degradation: Characterization, efficiency and mechanism. Chemical Engineering Journal 416 (2021) 128118.

[5] L. Yang, X. Ren, Y. Zhang, Z. Chen. Heterogeneous activation of peroxymonosulfate by Cu^+-decorated g-C_3N_4 under sunlight for degradation of organic pollutants. Journal of Environmental Chemical Engineering 9 (2021) 106596.

[6] Z. Wang, Y. Xu, C. Wang, L. Yue, T.Liu, Q. Lan, X. Cao, B. Xing. Photocatalytic inactivation of harmful algae Microcystis aeruginosa and degradation of microcystin by g-C_3N_4/Cu-MOF nanocomposite under visible light. Separation and Purification Technology 313 (2023) 123515.

[7] F. Guo, W. Shi, M. Li, Y. Shi, H. Wen. 2D/2D Z-scheme heterojunction of $CuInS_2$/g-C_3N_4 for enhanced visiblelight-driven photocatalytic activity towards the degradation of tetracycline. Separation and Purification Technology 210 (2019) 608–615.

[8] M.B. Nguyen, P.T. Lan, N.T. Anh, N.N. Tung, S. Guan, V.P. Ting, T.T.B. Nguyen, H.V. Doan, M.T. Tung, T.D. Lam. Ternary heterogeneous Z-scheme photocatalyst TiO_2/$CuInS_2$/OCN incorporated with carbon quantum dots (CQDs) for enhanced photocatalytic degradation efficiency of reactive yellow 145 dye in water. RSC advance, 2023, 13, 35339.

Table S2 Possible intermediates of LVF during the reaction

Name	m/z	Structure
LVX	m/z=362	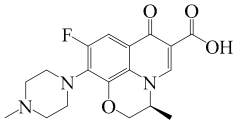
L1	m/z=393	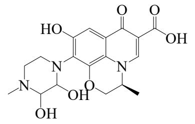
L2	m/z=290	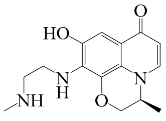
L3	m/z=273	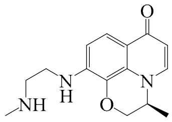
L4	m/z=229	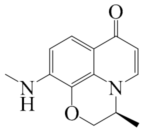
L5	m/z=301	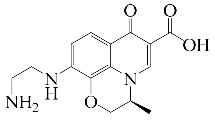
L6	m/z=239	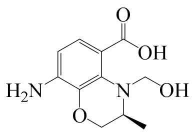
L7	m/z=318	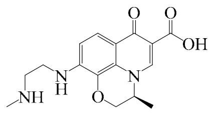

L8	m/z=261	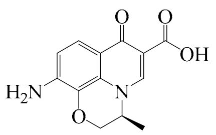
L9	m/z=378	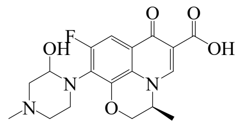
L10	m/z=246	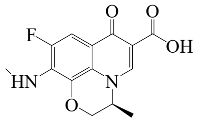

Table S3 Studies on of levofloxacin (LVF) degradation on various catalysts

No.	Systems	Light source	Catalyst	Reaction time and efficiency	Operation parameters
1	Vis+PMS+ $SrCoO_3/MnFe_2O_4/MoS_2$	300 W Xe lamp	MoS_2 embedded manganese ferrite doped perovskite	20 min; 95.1%	LVF = 10 mg/L, 100 mL, catalysts = 0.1 g/L, PMS = 0.5 g/L, stirring speed = 300 rpm, T = 25 ◦C.
2	Vis+PMS+Co_3O_4/RM	125 W high-pressure mercury lamp coated with fluorescent powder	waste red mud-supported Co_3O_4 quantum dots	20 min; 95.3%	$[PMS]_0$ = 1.0 mM, $[Co_3O_4/RM\text{-}1200]_0$ = 0.1 g/L, $[LVF]_0$ = 10 mg/L.
3	Vis+PMS+Co-Fe-CN	400 W halogen lamp	Co, Fe co-doped g-C_3N_4 composites	50 min; 92.1%	[LVFX] = 20 mg/L, [photocatalyst] = 0.4 g/L, [PMS] = 1.0 mM, initial pH = 6.8, T = 25 ± 1 ◦C
4	Vis+PMS+$Bi_2O_3/BiFeO_3$	350W cold light source xenon lamp	$Bi_2O_3/BiFeO_3$	60 min; 91.2%	pH = 7; catalyst dosage 0.5 g/L; PMS concentration 0.4 g/L
5	Vis+PMS+SVs-In_2S_3/TiO_2	Visible light	Internal electric field-mediated sulfur vacancies-modified-In_2S_3/TiO_2 thin-film heterojunctions	60 min; 92.4%	LVF=10mg/L; PMS=0.5 mM;
6	Vis+PMS+CN/BVO/CFO	500 W Xe lamp	Magnetic dual Z-scheme g-$C_3N_4/BiVO_4/CuFe_2O_4$ heterojunction	60 min; 96.2%	[LEV]0 = 10 mg·L^{-1}, [catalyst] = 200 mg L^{-1}, [PMS] = 1 mM, pH = 7

7	Vis+PMS+10%Ag/AgCl@ZIF-8 to g-C_3N_4	150 W Xe lamp	Ag/AgCl@ZIF-8 modified g-C_3N_4 composite	60 min; 87.3%	[catalyst] = 1.0 g/L, [PMS] =2.0 mM, [LVFX] = 0.01 g/L, pH = 6.5.
8	Bi_2O_3/$CuBi_2O_4$/Vis/PMS	Xe lamp	Z-scheme Bi_2O_3/$CuBi_2O_4$ heterojunction	120 min; 70.62%	LVF concentration: 13 mg/L; pH=7; catalyst dosage= 0.6 g/L; PMS concentration=0.5g/L
9	Vis+PMS+ZIF-CFO/FO	400 W halogen lamp	ZIF-derived $CoFe_2O_4$/Fe_2O_3 combined with g-C_3N_4	40 min; 95.1%	[LVFX] = 20 mg/L, [photocatalyst] = 0.4 g/L, [PMS] = 1.0 mM, initial pH = 7.2, and T = 25 ± 1 °C

[1] Y. He, J. Qian, P. Wang, J. Wu, B. Lu, S. Tang, P. Gao. Acceleration of levofloxacin degradation by combination of multiple free radicals via MoS_2 anchored in manganese ferrite doped perovskite activated PMS under visible light. Chemical Engineering Journal 431 (2022) 133933.

[2] Q. Li, G. Wei, L. Zhang, Z. Li, J. Li. Activation of peroxymonosulfate by a waste red mud-supported Co_3O_4 quantum dots under visible light for the degradation of levofloxacin. Chemical Engineering Journal 452 (2023) 139382.

[3] P. Guo, X. Hu. Co, Fe co-doped g-C_3N_4 composites as peroxymonosulfate activators under visible light irradiation for levofloxacin degradation: Characterization, performance and synergy mechanism. Colloids and Surfaces A: Physicochemical and Engineering Aspects 648 (2022) 129423.

[4] L. Liu , Y. Li, C. Zhu , J. Zhang , L. Chen. Degradation of levofloxacin hydrochloride by Bi_2O_3/$BiFeO_3$ activated peroxymonosulfate driven by visible light. Optical Materials 143 (2023) 114200.

[5] C. Ding, Y. Lu, J. Guo, W. Gan, S. Qi, Z. Yin, M. Zhang, Z. Sun. Internal electric field-mediated sulfur vacancies-modified-In_2S_3/TiO_2 thin-film heterojunctions as a photocatalyst for peroxymonosulfate activation: Density functional theory calculations, levofloxacin hydrochloride degradation pathways and toxicity of intermediates. Chemical Engineering Journal 450 (2022) 138271.

[6] X. Li, T. Chen, Y. Qiu, Z. Zhu, H. Zhang, D. Yin. Magnetic dual Z-scheme g-C_3N_4/$BiVO_4$/$CuFe_2O_4$ heterojunction as an efficient visible-light-driven peroxymonosulfate activator for levofloxacin degradation. Chemical Engineering Journal 452 (2023) 139659.

[7] J. Zhou, W. Liu, W. Cai. The synergistic effect of Ag/AgCl@ZIF-8 modified g-C_3N_4 composite and peroxymonosulfate for the enhanced visible-light photocatalytic degradation of levofloxacin. Science of the Total Environment 696 (2019) 133962.

[8] L. Liu , Y. Li, C. Zhu , N. Yang , Y. Li , F. Su , J. Qian. Visible light-driven Z-scheme Bi_2O_3/$CuBi_2O_4$ heterojunction with dual metal ions cycle for PMS activation and Lev degradation. Inorganic Chemistry Communications 158 (2023) 111531.

[9] P. Guo, X. Hu. ZIF-derived $CoFe_2O_4$/Fe_2O_3 combined with g-C_3N_4 as high-efficient photocatalysts for enhanced degradation of levofloxacin in the presence of peroxymonosulfate. Journal of Alloys and Compounds 914 (2022) 165338.
